# Spinal cord infarction following epidural and general anesthesia: a case report

**DOI:** 10.1186/s40981-017-0109-2

**Published:** 2017-08-16

**Authors:** Kaori Kobayashi, Noriko Narimatsu, Takafumi Oyoshi, Takashi Ikeda, Toshimitsu Tohya

**Affiliations:** 1 Department of Anesthesiology, Kumamoto Rosai Hospital, 1670, Takehara-machi, Yatsushiro, Kumamoto Japan; 2 Department of Orthopedic Surgery, Kumamoto Rosai Hospital, Yatsushiro, Japan; 3 Department of Obstetrics and Gynecology, Kumamoto Rosai Hospital, Yatsushiro, Japan

**Keywords:** Spinal cord infarction, Epidural anesthesia, Magnetic resonance imaging

## Abstract

**Background:**

Epidural anesthesia is widely used for postoperative analgesia and rarely causes permanent neurological complications. We report a case of paraplegia following abdominal surgery under combined epidural/general anesthesia.

**Case presentation:**

A 75-year-old woman underwent a scheduled abdominal total hysterectomy and bilateral salpingo-oophorectomy for suspected endometrial cancer. In the operating room, an epidural catheter was inserted at T11/12 while the patient was conscious. The needle entered smoothly, with no observed bleeding, paresthesia, or pain, and general anesthesia was induced. During surgery, 4 mL of 0.25% levobupivacaine and 0.1 mg of fentanyl were administered via the epidural catheter, and a solution of 2.5 μg/mL fentanyl and 0.2% levobupivacaine was continuously infused at 4 mL/h for postoperative analgesia. The patient promptly regained consciousness and could move her bilateral lower extremities without difficulty upon leaving the operating room. During the first postoperative night, she complained of an absence of sensation and weakness in the lower extremities. By the morning of the second postoperative day, she had developed paralysis and sensory losses associated with touch, temperature, pinprick, and vibration below T5. The epidural infusion was stopped. Magnetic resonance imaging (MRI) revealed a hyperintense area of the thoracic cord from T8 to T11, and spinal cord infarction was suspected. Ossification of the yellow spinal ligaments between T11 and T12, resulting in thoracic canal stenosis and thoracic spinal cord compression, were observed. Notably, the epidural catheter was inserted at the same site where the thoracic canal stenosis was present.

**Conclusions:**

Permanent neurological complications of epidural anesthesia are rare. Studies of neurological complications after epidural/spinal anesthesia have noted the possibility of spinal anomalies, such as lumbar stenosis, in relation to neurological complications after epidural/spinal anesthesia. In this case, the onset of spinal cord infarction may have occurred coincidentally with catheter insertion into the site of existing spinal stenosis. Therefore, it is important to evaluate lower extremity symptoms and consider spinal disease before administering epidural anesthesia. Spinal cord infarction may be prevented by preoperatively identifying spinal lesions using computed tomography or MRI in cases of suspected spinal disease.

## Background

Although epidural anesthesia is widely used for postoperative analgesia and rarely causes permanent nerve complications, some patients develop subsequent paralysis. Here we report a case of spinal cord infarction in a patient who developed paraplegia and sensory impairment after surgery under combined epidural and general anesthesia.

## Case presentation

The patient was a 75-year-old woman who had been taking oral medication for underlying hypertension. Prior to surgery, she could independently perform activities of daily living without experiencing symptoms, such as numbness in the lower extremity. She was scheduled to undergo an abdominal total hysterectomy and bilateral salpingo-oophorectomy for suspected endometrial cancer. After admission to the operating room, an epidural catheter (Perifix®, B. Braun, Germany) was inserted at T11/12 with the patient in a lateral position using a para-median approach with an 18-gauge Tuohy needle. A loss of resistance with normal saline was used to confirm needle advancement into the epidural space. The distance from the skin to the epidural space was 6 cm, and a catheter was inserted 5 cm toward the epidural space. The needle entered smoothly, with no observed bleeding, paresthesia, or pain. General anesthesia was induced with 0.25 μg/kg/min remifentanil, 50 mg of propofol and 40 mg of rocuronium and maintained with sevoflurane and remifentanil. The trachea was intubated with an endotracheal tube.

Vasoconstrictors, such as ephedrine and phenylephrine, were required to maintain an intraoperative systolic blood pressure of 80–90 mmHg. During surgery, 4 mL of 0.25% levobupivacaine and 0.1 mg of fentanyl were administered via the epidural catheter. The total operative duration was 80 min, and the total anesthesia duration was 123 min. For postoperative analgesia, the patient received a continuous 4 mL/h infusion of a 2.5 μg/mL fentanyl and 0.2% levobupivacaine solution.

**Fig. 1:**
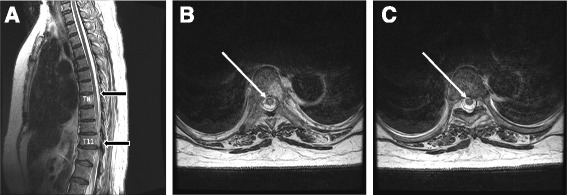
**a** Sagittal magnetic resonance imaging (MRI) revealed hyperintensity in the thoracic cord from T8 to T11 (*arrows*); spinal cord infarction was suspected. **b** Axial MRI of the T8 spinal cord level. A hyperintense area was apparent (*white arrow*). **c** Axial MRI of the T6 spinal cord level. Normal intensity was observed (*white arrow*)

**Fig. 2:**
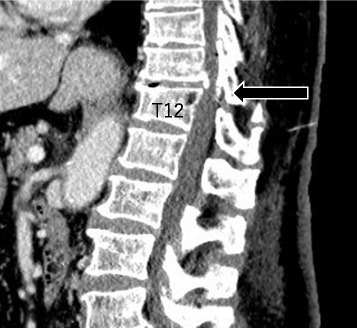
Sagittal computed tomography (CT) image of the vertebrae. The tip of the epidural tube was present at narrowest area of the spine (*arrow*)

**Fig. 3:**
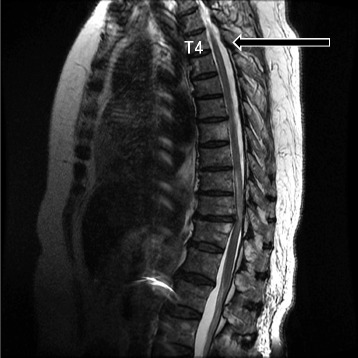
One week after the operation, sagittal MRI revealed that the hyperintense region had extended to approximately the T4 level

**Fig. 4:**
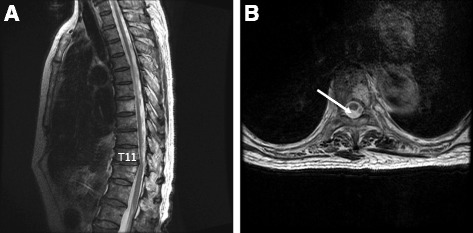
**a** Sagittal MRI on postoperative day 93 demonstrated that the hyperintense region was localized to T11. **b** Axial MRI of the T8 spinal cord level. Scarring of the infarction occurred, and the fluid space was extended

The patient promptly regained consciousness and was transferred from the operating room following extubation. She could move her bilateral lower extremities without difficulty when leaving the operating room. During the afternoon of the first postoperative day, she could stand without numbness or weakness in the lower extremities. On the night of the same day, the patient complained of a loss of sensation and weakness in the bilateral lower extremities. Her lower extremity symptoms failed to resolve by the following morning (second postoperative day), at which point sustained-release epidural administration was stopped. However, the patient developed paralysis and a loss of touch, temperature, pinprick, and vibration sensations below the T5 level. In addition, her lower extremities were flaccid. To determine whether an epidural hematoma had occurred in her epidural space, a magnetic resonance imaging (MRI) examination was immediately performed. The MRI revealed the following: (1) There were no epidural masses, i.e., no epidural hematomas. (2) The patient exhibited yellow ligament ossification. (3) T2-weighted MRI revealed hyperintensity in the T8-T11 spinal cord region (Fig. 1a–c). In addition, a computed tomography (CT) examination revealed that the epidural catheter had been inserted at the same site where the thoracic canal stenosis was most severe (Fig. 2). Therefore, she was diagnosed with a spinal cord infarction. Other spinal diseases (e.g., spinal degenerative disease) were excluded because of the rapid onset. Spinal cord infarctions are usually treated conservatively, as surgical interventions for this disease are inadequate. She was managed conservatively. She was treated with blood pressure control therapy and rehabilitation consisting of muscle training and walking practice, among other exercises. The epidural catheter was removed on the third postoperative day. One week later, an MRI examination revealed that the hyperintense region had extended superiorly to the T4 level (Fig. 3). The patient underwent rehabilitation and regained the ability to move her lower extremities on postoperative day 30. Although the patient could stand on her left leg, she was unable to stand on her right leg. She received continuous bladder catheterization, and her defecation was controlled by drugs. An MRI examination on postoperative day 93 demonstrated that the hyperintense region was localized to T11, where the thoracic canal stenosis was originally present and the epidural catheter was inserted (Fig. 4a, b). On postoperative day 286, the patient was transferred to a rehabilitation hospital.

## Discussion

We are reporting a case of spinal cord infarction that occurred after epidural anesthesia. There are few similar case reports in the English literature. Spinal cord infarction accounts for only 0.3–1% of stroke cases and therefore has a low incidence relative to other types of stroke [1]. Some studies have reported that age, hypertension, diabetes, obesity, a history of cerebral infarction, and atherosclerotic lesions are risk factors for spinal cord infarction [1, 2]. Other studies have reported the roles of aortic disease and related surgical treatments, as well as spinal disease and related surgical treatments, as risk factors for spinal cord infarction [3, 4]. Although epidural anesthesia is widely used for intraoperative and postoperative analgesia, it can lead to several complications, of which permanent nerve damage is the most severe. Epidural hematoma after epidural anesthesia is a typical nerve complication. While spinal cord infarction as observed in this case is rare, it should still be taken into consideration.

Concerning the cause of spinal cord infarction after anesthesia and surgery, there are few reports in the literature. Some reports have assumed that spinal cord ischemia following epidural anesthesia was associated with intraoperative hypotension [5, 6], toxicity from local anesthesia [5], or vasospasm related to the administration of a local epinephrine-containing anesthetic [5–7]. However, no clear associations have been demonstrated in either case. Another study noted that in elderly patients and those with abnormal spine anatomy, an excessive anesthetic agent volume may cause spinal cord compression [8]. Furthermore, a study of neurological complications after epidural and spinal anesthesia estimated that spinal cord infarction occurred in approximately five per million patients [9]. Four of these five patients were reported to have anatomical abnormalities, such as lumbar spine stenosis and subarachnoid cysts. Therefore, the authors suggested that these spinal abnormalities are associated with neurological complications after epidural/spinal anesthesia [9]. One prior case report showed that spinal cord infarction developed due to severe disc herniation without anesthesia or surgery [10]. Thus, compression to the spinal cord may be an important factor. In our case, the spinal cord infarction occurred at the narrowest area of the spinal cord. This area was narrowed due to the ossification of the yellow ligament and became narrower by the tip of the epidural puncture tube. The small vessels around the narrow spinal cord might have been compressed, causing the infarction. Therefore, patients who complain of numbness and/or weakness of the lower limbs, intermittent claudication, etc. should undergo preoperative MRI examinations of the spinal cord. Some studies have examined paralysis after aortic surgery [11]. The patient in our case underwent soft tissue surgery in the lower abdomen, and the relationship between this abdominal surgery and the patient’s spinal cord infarction may have been negative. In our patient, the causal relationship between epidural anesthesia and spinal cord infarction is still not clear. However, it is unique that her clinical symptoms occurred after anesthesia and surgery. Based on this presentation, anesthesiologists and surgeons must recognize that spinal cord infarction may occur after combined general and epidural anesthesia or surgery.

Concerning the treatment of spinal cord infarction, conservative treatments are usually chosen. In this patient, the motor and sensory deficits were clinically distinct, indicating the infarction of three spinal arteries (one anterior and two posterior). A surgical intervention would have been difficult, and the standard drug therapy is aspirin, based on the consensus recommendation for the acute treatment of ischemic stroke at any site. However, no direct studies have examined the efficacy of drug therapy in spinal cord infarction.

Yellow ligament ossification frequently occurs in middle-aged people and is thought to particularly affect the lower thoracic spine because it often occurs at T10/11 and T11/12 [12]. Numbness and pain in the lower extremities are common symptoms of this condition. In this case, MRI revealed ossification of the yellow ligaments at the T11/12 spinal level, with the accompanying findings of thoracic spinal cord compression. The site of spinal cord infarction was also consistent with the location of epidural catheter insertion. Although the patient in this case did not present with clinical symptoms of thoracic canal stenosis, such as numbness or weakness of the lower extremities, prior to surgery, the onset of spinal cord infarction may have occurred simultaneously with the insertion of the catheter into a site where spinal stenosis was already present.

A preoperative examination with a focus on yellow ligament ossification may be necessary in middle-aged patients undergoing lower abdominal surgery with epidural anesthesia. A preoperative plain chest radiography evaluation of spinal ossification and an evaluation of lower extremity symptoms may be important for reducing the incidence of spinal cord infarction related to epidural anesthesia. When administering epidural anesthesia to patients with a spinal disease, lower thoracic spine catheterization might have to be avoided. Spinal cord infarction in cases of suspected spinal disease may be prevented by preoperatively identifying spinal lesions via a CT or MRI evaluation.

## Conclusions

We report a case of paraplegia following post-epidural analgesia in a patient diagnosed with spinal cord infarction. MRI revealed spinal cord compression resulting from yellow ligament ossification; notably, the site of spinal cord infarction was consistent with the location of epidural catheter insertion. Spinal cord infarction may be prevented by preoperatively identifying spinal lesions using CT or MRI prior to epidural anesthesia when spinal disease is suspected.

## Abbreviations

CT: Computed tomography
MRI: Magnetic resonance imaging
